# RNA Sequencing (RNA-Seq) Based Transcriptome Analysis in Immune Response of Holstein Cattle to Killed Vaccine against Bovine Viral Diarrhea Virus Type I

**DOI:** 10.3390/ani10020344

**Published:** 2020-02-21

**Authors:** Bryan Irvine Lopez, Kier Gumangan Santiago, Donghui Lee, Seungmin Ha, Kangseok Seo

**Affiliations:** 1 Division of Animal Genomics and Bioinformatics, National Institute of Animal Science, Rural Development Administration, Wanju 55365, Korea; irvinelopez@korea.kr; 2 Department of Animal Science and Technology, Sunchon National University, Suncheon 57922, Korea; santiagokier2015@gmail.com (K.G.S.); a3832737@naver.com (D.L.); 3 Department of Animal Science, College of Agriculture, Central Luzon State University, Science City of Muńoz 3120, Philippines; 4 Dairy Science Division, National Institute of Animal Science, Rural Development Administration, Cheonan 31000, Korea; justusha@korea.kr

**Keywords:** Bovine Viral Diarrhea Virus, RNA-Seq, Transcriptome analysis, Holstein cattle

## Abstract

**Simple Summary:**

Due to the undeniable detrimental impact of bovine viral diarrhea virus (BVDV) on cattle worldwide, various preventive approaches are carried out to control the spread of this disease. Among the established preventive approaches, vaccination remains the most widely used cost-effective method of control. Hence, a deeper study into the host immune response to vaccines will further refine the efficacy of these vaccines; the identification of differentially expressed genes (DEGs) related to immune response might bring a long-lasting solution. Thus far, studies showing the genes related to the immune response of cattle to vaccines are still limited. Therefore, this study identified DEGs in animals with high and low sample to positive (S/P) ratio based on the BVDV antibody level, using RNA sequencing (RNA-seq) transcriptome analysis, and functional enrichment analysis in gene ontology (GO) annotations and the Kyoto Encyclopedia of Genes and Genomes (KEGG) pathway. Results revealed that several upregulated and downregulated genes were significantly annotated to antigen processing and presentation (MHC class I), immune response, and interferon-gamma production, indicating the immune response of the animals related to possible shaping of their adaptive immunity against the BVDV type I. Moreover, significant enrichment to various KEGG pathways related to the development of adaptive immunity was observed.

**Abstract:**

Immune response of 107 vaccinated Holstein cattle was initially obtained prior to the ELISA test. Five cattle with high and low bovine viral diarrhea virus (BVDV) type I antibody were identified as the final experimental animals. Blood samples from these animals were then utilized to determine significant differentially expressed genes (DEGs) using the RNA-seq transcriptome analysis and enrichment analysis. Our analysis identified 261 DEGs in cattle identified as experimental animals. Functional enrichment analysis in gene ontology (GO) annotations and Kyoto Encyclopedia of Genes and Genomes (KEGG) pathways revealed the DEGs potentially induced by the inactivated BVDV type I vaccine, and might be responsible for the host immune responses. Our findings suggested that inactivated vaccine induced upregulation of genes involved in different GO annotations, including antigen processing and presentation of peptide antigen (via MHC class I), immune response, and positive regulation of interferon-gamma production. The observed downregulation of other genes involved in immune response might be due to inhibition of toll-like receptors (TLRs) by the upregulation of the Bcl-3 gene. Meanwhile, the result of KEGG pathways revealed that the majority of DEGs were upregulated and enriched to different pathways, including cytokine-cytokine receptor interaction, platelet activation, extracellular matrix (ECM) receptor interaction, hematopoietic cell lineage, and ATP-binding cassette (ABC) transporters. These significant pathways supported our initial findings and are known to play a vital role in shaping adaptive immunity against BVDV type 1. In addition, type 1 diabetes mellitus pathways tended to be significantly enriched. Thus, further studies are needed to investigate the prevalence of type 1 diabetes mellitus in cattle vaccinated with inactivated and live BVDV vaccine.

## 1. Introduction

Bovine viral diarrhea virus (BVDV) is an economically important pathogen of domestic and wild ruminants affecting multiple organ systems, incurring most economic losses due to respiratory diseases, low reproductive performance (due to reduced conception rates), early embryonic deaths, abortion, congenital weak calves, and high costs of control programs [1,2,3,4]. BVDV belongs to the genus *Pestivirus* within the family *Flaviviridae*, with two species, namely BVDV1 and BVDV2; both consist of strains, belonging to biotypes non-cytopathogenic or cytopathogenic, based on cell-cultured characteristics [5]. The non-cytopathogenic strain has the ability to cross the maternal placenta, infecting the growing fetus at early gestation (before 150 gestation days) due to the undeveloped immune system and failure of recognizing the virus as foreign [6]. This fetal infection affects fetal development [7], and results in persistently infected born calves, shedding lifelong reservoir of BVDV in the herd, while cytopathic BVDV plays a vital role by superinfecting persistently infected cattle, leading to mucosal disease [5]. In addition, recent studies reported that the immunosuppressive ability of BVDV heightens other viral disease potentiators, particularly the bovine respiratory disease [8,9,10]. The differences in genotypes and biotypes of BVDV, a wide range of susceptible hosts, the ability to induce persistent infection, and intervene with both innate and adaptive immunity, makes the prevention and control program difficult [4].

To date, modified live viral and inactivated viral vaccines are widely used to prevent the consequences of BVDV infection [11]. However, vaccine efficacy varies, depending on the animal’s nutritional status [12], maternal antibody from the dam colostrum [13], and the presence of persistently infected cattle in the herd. Thus far, numerous studies were conducted to improve vaccine efficacy against BVDV, yet still remains prevalent among the cattle herd worldwide.

Numerous studies focusing on the transcriptomic analysis of animals infected with various diseases were carried out. In the study of Li et al. [4], various differentially expressed genes (DEGs) related to goat immune response, including inflammation, defense response, cell locomotion, and cytokine/chemokine-mediated signaling were revealed by transcriptome analysis with samples from BVDV2 artificially infected goat peripheral blood mononuclear cells (PBMCs). Similar methodology was done in the study of Singh et al. [14], where various significant DEGs related to the immune system processes of goat and sheep against bluetongue virus serotype 16 (BTV-16) were revealed, such as NFκB, MAPK, Ras, NOD, RIG, TNF, TLR, JAK-STAT, and VEGF signaling pathways. Meanwhile, comparative transcriptomic analyses between infected and non-infected animals were conducted by Barreto et al. [15], where infected bovine were observed to have massive changes in the expression profiles of keratinocyte, immune system, cell proliferation, and apoptosis genes. 

All of these studies used transcriptome analysis and next-generation sequencing (NGS) approach particularly the RNA sequencing (RNA-Seq). RNA sequencing is a developed method that uses deep sequencing technology for transcriptome profiling [16]; aside from providing a comprehensive picture of the transcriptome, it also reveals the activity and mechanism of the molecular structure and explores the biological function of a gene [17]. Furthermore, future works using this technology are considered, such as genetic linkage mapping, quantitative trait analysis, disease-resistant strains, effective vaccines, and therapies development [18].

Although genotyping and RNA sequencing still remain costly, such studies may provide novel insights and solid foundation in improving herd performance through the robustness of animals against viral diseases. However, studies pertaining to the immune responses of the animal to the vaccine were not fully explored. Thus, the objective of this study is to identify differentially expressed genes (DEGs) related to the functional immune response of the host vaccinated with the inactivated BVDV type I vaccine, and to provide insight on how vaccines improve the immunity of animals against diseases. 

## 2. Materials and Methods 

### 2.1. Experimental Animals and Vaccination

A total of 107 vaccinated Holstein Cattle (*Bos taurus)* was used in the study. Multivalent killed vaccine Bar Vac Elite 4-HS (Boehringer Ingelheim Vetmedica, Inc., St Joseph, MO, USA) containing antigen of infectious bovine rhinotracheitis virus, bovine viral diarrhea virus (BVDV, type I), bovine respiratory syncytial virus (BRSV), Myxovirus parainfluenza type 3 (PI3) and *Haemophilus somnus* bacterin were given intramuscularly, as prescribed by the manufacturer. 

Blood samples were collected from the jugular vein at 7, 28, and 168 d post-vaccination. Subsequently, blood was allowed to coagulate for 1–2 h at 4 °C and centrifugate for 20 min at room temperature with the relative centrifugal force of 1800 × g. Serum was collected and aliquoted into 1.5 mL tubes and stored below −60 °C until ELISA test. 

### 2.2. Serological Antibody Detection

Competitive ELISA, using VDPro BVDV AB ELISA (Median Diagnostics Inc., Chuncheon, Republic of Korea) was used to assay the antibody responses of each animal against the vaccine. Assaying was performed as per manufacturer protocols. Concisely, the BVDV gp63 antigen was allowed to absorb in the polystyrene plate and bind with antibodies in serum samples. It was competed for corresponding hydrogen peroxide conjugated monoclonal antibodies. The chromogenic change after the addition of 3, 3’, 5, 5’-Tetramethylbenzidine substrate was measured at 450 nm optical density using BioTek ELISA reader and Gen5 2.07 software; results with lower color development signify a higher level of antibody. The optical density (OD) value was measured using a microplate reader set at 405 nm and concentration was valued with the corresponding standard references. The obtained OD value of BVDV type I antibodies were evaluated for the comparative value, related to the positive control value, to get antibodies level in the sample to positive (S/P) ratio form by applying the equation as below.

(1) $$\frac{S}{P}=\frac{\mathrm{Sample}\;O.D.\;\mathrm{value}\;-\;\mathrm{Negative}\;\mathrm{Control}\;O.D.\;\mathrm{value}}{\mathrm{Positive}\;\mathrm{control}\;O.D.\;\mathrm{value}\;-\;\mathrm{Negative}\;\mathrm{Control}\;O.D.\;\mathrm{value}}$$

**Figure 1:**
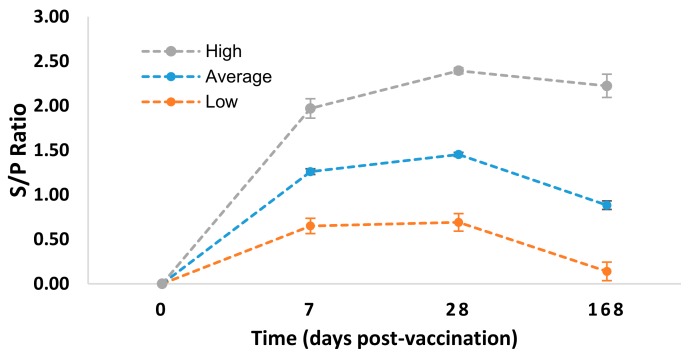
Sample to positive (S/P) ratio of cattle groups identified as high (*n* = 5), low (*n* = 5) and average (*n* = 107) bovine viral diarrhea virus (BVDV) type I antibody level at different time points. The error bars indicate standard error.

The obtained BVDV type I S/P ratio, and other immune-related parameters, such as TNF-alpha, IFN-gamma, IL-17A, IL-1b, IL-4, IL-2, and IL-6 were used as the basis for selecting experimental animals. Among the 107 experimental animals, only ten (10) animals were selected and grouped into two groups, namely the low and high BVDV type I groups; each group had five (5) animals (Figure 1).

### 2.3. RNA Isolation, Library Preparation, and RNA Sequencing (RNA-seq)

Total RNA was stabilized and isolated from the blood samples (collected after vaccination) of the selected animal groups using Tempus Blood RNA Tube (Applied Biosystems, Seoul, Korea), according to the manufacturer’s instructions. RNeasy MinElute Cleanup Kit (Qiagen, Valencia, CA, USA) was used to purify and concentrate the previously isolated RNA. RNA quality based on RNA integrity number (RIN) was determined using Agilent Technologies 2100 Bioanalyzer (Agilent, Santa Clara, CA, USA) with an acceptable RIN value of ≥7. These RNA samples were used to generate RNA-Seq transcriptome libraries, using the TruSeq Stranded Total RNA LT sample preparation kit (Globin) of Illumina (San Diego, CA, USA). To ensure the quality of prepared libraries, the size of the PCR enriched fragments were verified by checking the template size distribution, by running on Agilent Technologies 2100 Bioanalyzer using a DNA 1000 chip, and were quantified using qPCR according to the Illumina qPCR quantification protocol guide. After a series of quality control and quantification, prepared paired-end libraries for animals with high (test) and low (control) BVDV type I antibody were then sequenced with the Illumina NovaSeq 6000 platform, performed by TNT Research Corporation Limited (Anyang, South Korea).

### 2.4. Reads Trimming, Mapping, and Assembly of Sequenced RNA Reads

Quality control (QC) of raw paired-end reads were done by trimming reads using Trimmomatic 0.38 (http://www.usadellab.org/cms/?page=trimmomatic). QC included the removal of adapter sequence, contaminant DNA, and low-quality reads with lengths below 36 bp. Clean reads (cDNA) were indexed to reference (*Bos taurus*) cattle genome GCF_002263795.1 ARS-UCD1.2 and were mapped against the reference genome using HISAT2 version 2.1.0 (Bowtie2 aligner) (https://ccb.jhu.edu/software/hisat2/index.shtml). Reference-based aligned read assembly of transcripts was performed using the StringTie 1.3.4d (https://ccb.jhu.edu/software/stringtie/). This allowed the identification of transcript or genes with annotation information in the assembled genome, while genes without annotated information were defined as new transcripts [19]. On the other hand, mapping of each sample without the –e option of StringTie allowed the prediction of novel transcript and novel alternative splicing transcript. The gffcompare program from GFF utilities was used to compare existing annotations and distinguish novel transcript types.

### 2.5. Differential Expression Genes (DEGs) Analysis and Clustering

The identification of DEGs between case and control samples was based on the expression level on each transcript, which was calculated using the fragments per kilobase of exon per million mapped reads (FPKM) method. The DESeq2 package, equipped with fold change and negative binomial (nbinom) Wald test, was used for differential expression analyses. DEGs were identified based on the following parameters: the logarithmic fold change was greater than or equal to 2 (|fc|>=2) and nbinom Wald test raw *p* < 0.05. In addition, hierarchical clustering of significant genes was done to determine the similarity level of each sample. 

### 2.6. Functional Annotation and Enrichment Analysis

DEGs were based on several functional annotation databases, specifically gene ontology (GO) (http://geneontology.org/) and Kyoto Encyclopedia of Genes and Genomes (KEGG) (http://kegg.jp). Enrichment analysis was performed using the Database for Annotation, Visualization and Integrated Discovery 6.8 (DAVID) tool (http://david.abcc.ncifcrf.gov/) equipped with the modified Fisher’s exact test. DEGs with a *p*-value of less than 0.05 were significantly considered enriched in GO terms and KEGG pathways. 

## 3. Results

### 3.1. Experimental Animals

Table 1 shows the two groups of experimental animals identified in this study, namely, low and high group, based on the level of BVDV type I antibody and level of immune responses, including TNF-alpha, IFN-gamma, IL-17A, IL-1b, IL-4, IL-2, and IL-6. For the earlier group, there were five identified animals namely (ID number), 13064, 13083, 13090, 14010, and 14017, while a similar number of animals belong to the latter group, namely, 14107, 15060, 15071, 15083, and 15094.

### 3.2. Transcriptome Sequencing Data 

An average of 9.0G bp (Table 1) raw data for each sample was obtained from paired-end transcriptome sequencing from the Illumina NovaSeq 6000 platform. Prior to further analysis, raw data were subject to quality control using Trimmomatic version 0.38. The trimmed results show that the total read bases, GC (%), and Q30 (%) of each sample have values ranging from 7.0 G to 11.0 G, 44.86% to 45.99%, and 94.65% to 95.31%, respectively, as shown in Table 1. Trimmed data were mapped against the reference genome (GCF_002263795.1 ARS-UCD1.2) using the HISAT2 program. Obtained mapped reads were then assembled using StringTie-e option version 1.3.4d, which resulted in a total of 100,685 transcripts and 39,127 genes successfully mapped against the reference genome. Thereafter, the removal of low-quality transcripts and genes was done, leaving only 10,000 transcripts and 35,000 genes for differentiation analysis. Furthermore, a total of 1452 novel transcripts, 13,060 novel splicing variants, and 4199 novel genes were identified using the StringTie software.

### 3.3. Differentially Expressed Genes

**Figure 2:**
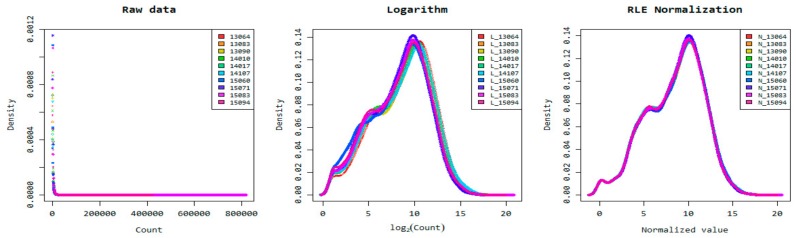
Density plot of normalized data using Relative Log Expression (RLE) normalization method based on read count and log2.

The resulting good quality genes from StringTie software were filtered by excluding genes with at least one zero count, leaving only 45% (16,315 genes) for DEG analysis. Prior to DEG analysis, expression levels between genes of each sample were first normalized using the Relative Log Expression (RLE) normalization method, based on raw read counts (Figure 2). 

**Figure 3:**
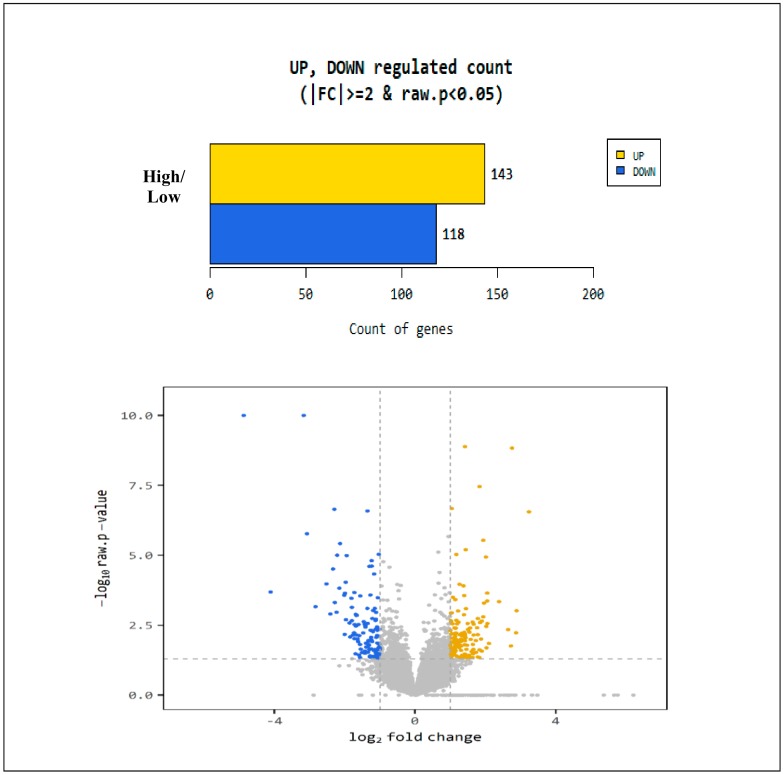
Number of up- and down-regulated genes after comparison of normalized values using the DESeq2 package.

The analysis of the differently expressed genes (DEGs) were done by comparing the normalized values using the DESeq2 package, equipped with log fold change and nbinom Wald test. A total of 261 genes were considered differentially expressed based on the threshold level (fold change (log2) ≥ 2 and *p*-value < 0.05) (Figure 3). Results of comparison analysis between animals with high (test) and low (control) BVDV type I antibody groups revealed that 143 genes were classified as up-regulated while the remaining 118 genes were down-regulated (Figure 3). 

### 3.4. Functional Enrichment Analysis of Identified DEGs

**Figure 4:**
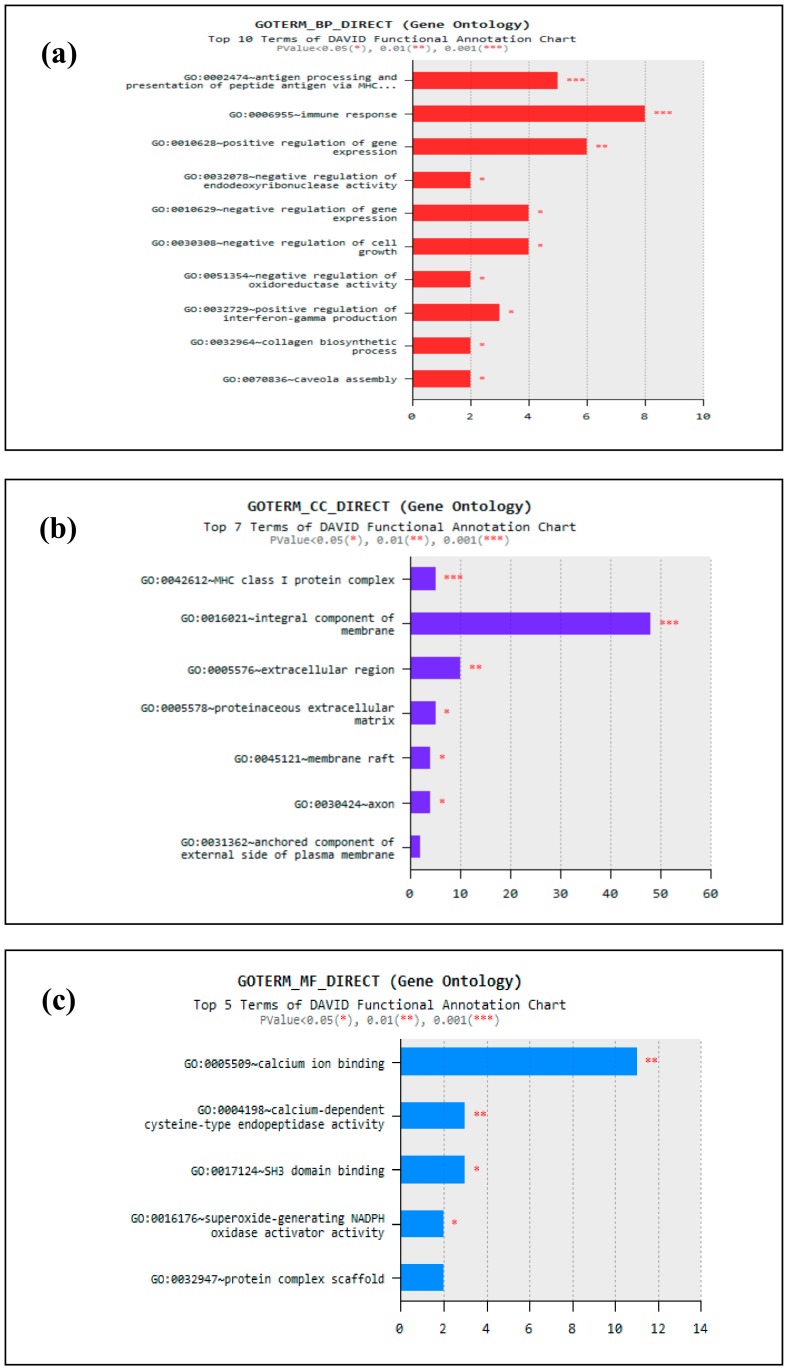
Gene ontology (GO) enrichment analysis of differentially expressed genes (DEGs) in vaccinated cattle, selected based on BVDV type I antibody level (**a**) GOTERM_Biological Process, (**b**) GOTERM_Cellular Component, and (**c**) GOTERM_Molecular Function. GO terms are located on the y-axis and terms with (*) (**) (***) means significant enrichment with a *p*-value of <0.05, 0.01, and 0.001, respectively.

RNA-seq transcriptome analysis successfully identify a total of 261 DEGs. Several functional annotation databases, such as gene ontology (GO) and KEGG pathway using the Database for Annotation, Visualization and Integrated Discovery 6.8 (DAVID) tool, were used to determine the biological function of these identified DEGs. DAVID gene enrichment analysis revealed 28 significant GO terms throughout the differentiation analysis (*p* < 0.05). However, there were only three GO major categories, namely biological process (GOTERM_BP), cellular component (GOTERM_CC), and molecular function (GOTERM_MF) where the significant DEGs were grouped based on their functionality (Figure 4). In this study, 38 significant DEGs were distributed to top 10 GOTERM_BP, namely antigen processing and presentation of peptide antigen (via MHC class I), immune response, positive regulation of gene expression, negative regulation of gene expression, negative regulation of cell growth, negative regulation of oxidoreductase activity, positive regulation of interferon-gamma production, collagen biosynthetic process, and caveola assembly. In GOTERM_MF, 21 DEGs were distributed into the following top 5 GO terms; calcium ion binding, calcium-dependent cysteine-type endopeptidase activity, SH3 domain binding, superoxide-generating Nicotinamide Adenine Dinucleotide Phosphate (NADPH) oxidase activator activity, and protein complex scaffold. Meanwhile, 78 DEGs were distributed to the top 7 GOTERM_CC as follows; integral component of membrane, class I protein complex, extracellular region, proteinaceous extracellular matrix, membrane raft, axon and anchored component of the external side of the plasma membrane. 

### 3.5. KEGG Pathway Enrichment Analysis

To allow deeper understanding of the biological function of significant DEGs, KEGG pathway enrichment analysis was done using DAVID 6.8 tool. Initially, KEGG pathways analysis successfully annotated DEGs into 10 pathways which later reduced into 5 significantly enriched pathways (*P<0.05*) namely; cytokine-cytokine interaction, platelet activation, ECM-receptor interaction, hematopoietic cell lineage, and ABC transporters (Table 2). Cytokine-cytokine interaction pathway involved 2 upregulated (IL18, IL1RAP) and 4 down-regulated DEGs (CCR8, CCL3, IL20RA, TGFB2); hematopoietic cell lineage pathway linked 4 upregulated DEGs (GP5, GP1BA, CD24, GP9); platelet activation pathway with 5 up-regulated DEGs (GP5, P2RX1, MAPK12, GP1BA, GP9); ECM-receptor interaction pathway with 3 up-regulated (GP5, GP1BA, GP9) and 1 down-regulated (ITGB4) DEGs and ABC transporters pathway with 1 upregulated (ABCB11) and 2 downregulated DEGs (LOC100296627, CFTR). Results obtained from GO and KEGG analysis indicated that various DEGs were involved in host immune response to inactivated BVDV Type I vaccine. 

## 4. Discussion

BVDV infection is undeniably detrimental to bovine raisers by reducing milk yield, and is associated with low reproductive performance and growth retardation, allowing the occurrence of other disease potentiators, premature culling, and a high rate of mortality to young stock [20]. Houe et al. [20] and Carman et al. [21] estimated that national herd could experience economic loss ranging between $10 million and $40 million per million calvings, and $40,000–$100,000 (USD) per herd, respectively. Thus, to prevent such negative effects of BVDV, the development of cost-effective controls, including vaccines and eradication schemes were considered [22]. However, despite effective control programs, BVDV infection remains rampant in most cattle herd worldwide. Evidence reveals that variability of the BVDV strains, cross placental ability of the virus leading to persistent infections, wide spectrum of susceptible hosts, and the ability to interfere both innate and adaptive immunity makes prevention and control, such as vaccination, less effective [4,23]. 

Recently, similar studies that used next-generation sequencing technology (NGS) purported various DEGs related to animal immune response against viral diseases, providing a deeper understanding of immune responses. Since the development of microarray-based analysis and completion of the Human Genome Project, more advanced sequencing technology has come about, such as RNA-Seq based transcriptome analysis [24]. Compared to DNA microarray-based technology, RNA–Seq provide greater dynamic range by directly revealing sequence identity crucial for annotation quantification of unknown genes and novel transcript isoforms [25,26]. In studies by Li et al., Singh et al., and Barreto et al. [4,14,15], RNA-Seq based transcriptome analyses were used to successfully identify both up- and down-regulated genes related to the host immune response during BVDV, bluetongue virus of sheep and goats, and bovine papillomatosis infection, respectively. 

In this study, cattle vaccinated with inactivated multivalent vaccine (BVDV type I, BRSV, Myxovirus parainfluenza type 3 (PI3), *Haemophilus somnus* bacterin) were evaluated days after the last vaccination, to identify and understand changes in gene expressions related to the immune response brought by the vaccine. Specifically, this study examines the only animal with a high and low BVDV type I antibody level; thus, DEGs identified in the transcriptome analysis were highly attributed to the immune response of the animal to inactivated BVDV type I vaccine. 

Vaccination is considered an effective tool in preventing and controlling infectious diseases involving the cooperative action of innate and adaptive immunity [27]. Innate immunity plays a key role in triggering adaptive immune response by involving hematopoietic cells, such as macrophages, mast cells, neutrophils, eosinophils, dendritic cells, natural killer cells, and non-hematopoietic cells, such as skin and epithelial linings of the gastrointestinal, genitourinary, and respiratory tract [28]. Meanwhile, adaptive immunity plays its vital role in the immune system as it involves a tightly regulated interaction between antigen -presenting cells and T and B lymphocytes that facilitate pathogen-specific immunologic effector pathways, immunologic memory, and regulation of host immune homeostasis [29].

In this study, functional enrichment analysis revealed upregulated DEGs related to both innate and adaptive immune responses, such as BoLA, IL18, and BCL3. Among identified immune-related genes, the bovine lymphocyte antigen (BoLA) caught the attention of the researcher, as it was directly involved in antigen presentation. The BoLA gene located on chromosomes BTA 23 [30], and generally known as the MHC of cattle, was reported to play an integral role in immune responsiveness and susceptibility to the diseases of the host animal [31]. MHC is a cell surface glycoprotein molecule, having the binding ability to foreign peptides, such as viral proteins, and provides context for the recognition of T-lymphocytes responsible for cell-mediated immunity [32,33]. In studies conducted by Gutierrez et al. [34] and Weigel et al. [35], it was discovered that MHC genes are strongly associated with disease resistance and susceptibility to a wide range of diseases; thus, it can be a natural strategy in controlling infectious diseases, by incorporating it to the selection index and in genetic manipulation techniques.

The IL18 and Bcl-3 gene was also identified, upregulated in this study; IL18 gene play an important role in the T-cell-helper type 1 (Th1) and are involved in the regulation of innate and adaptive immune response by inducing IFN-gamma in natural killer cells (NKC) and T helper (Th1) lymphocytes [36,37]. Primary precursors of IL-18 are expressed in epithelial cells of the body, while the primary sources are macrophages and dendritic cells [38]. In a study conducted on laboratory mice, IL-18 was considered an effective adjuvant by enhancing immunogenicity through its relevant activities, such as activator of NK cells, a strong stimulator of Th1 responses, and other immunoactive cytokines in Th1 cells, monocytes, and NK Cells [38]. 

Whereas, the BCL3 is a proto-oncogene member of the IkB family, and also reportedly plays an important role in immune responses. In a study by Schwarz et al., it was purported that the Bcl-3 protein interacts specifically with the NFkB subunits (p50 and p52). It was also reported that mice lacking the Bcl-3 gene exhibit normal development and immunoglobin levels, but the humoral immune response was severely affected, and fail to produce antigen-specific antibodies [39]. Further, in the study of Fredericksen et al. [40], it was observed that the BVDV-1 infected Madin-Darby bovine kidney cell line induces immune marker production, such as BCL3, IL-1, IL-8, IL-15, IL-18, Mx-1, IRF-1, and IRF-7 through the NF-kB signaling pathway. Furthermore, Carmody et al. [41] reported that Bcl-3 limits the strength of toll-like receptors (TLRs) that are responsible for triggering inflammatory cytokines production and development of both adaptive and innate immunity through p50 subunit ubiquitination stabilization. Thus, this limitation of TLR responses might be responsible for the downregulation of other DEGs related to inflammatory responses. 

Enrichment analysis through KEGG pathways of DEGs was done to understand signal transduction pathways activated and repressed by inactivated antigen (vaccine). Results of KEGG pathway enrichment analysis revealed five (5) significantly enriched pathways, such as platelet activation, cytokine-cytokine receptor interaction, ECM receptor interaction, hematopoietic cell lineage, and ABC transporters. Among identified significant pathways, cytokine-cytokine receptor interaction involved the most number of DEGs. The cytokine-cytokine (c-c) receptor interaction plays a vital role in health during immunological and inflammatory responses to diseases through the synergistic convergence of signaling pathways and divergence of the cytokine signal, which activates another cytokine system [42]. In this study, six (6) significant DEGs under c-c receptor interaction pathways were identified, namely; CCR8, CCL3, IL20RA, TGFB2, IL18, and IL1RAP with only the last two (2) DEGs identified as upregulated (IL18, IL1RAP). Downregulation of other DEGs belonging to c-c receptor interaction and linked to TLR may be attributed to previously reported upregulation of Bcl-3, which limits the duration of TLR responses that control deleterious inflammatory diseases. However, further studies are warranted to fully support this claim. 

Another significantly enriched pathway observed in this study is the extracellular matrix (ECM) receptor interaction pathway, which includes four (4) upregulated DEGs, namely, glycoprotein (GpV), GpIba, GpIX, and ITGB4. Briefly, ECM is a non-cellular component found in all tissue and organs, providing cellular constituents its physical framework, and it also plays a vital role in tissue morphogenesis, differentiation, and homeostasis by initiating crucial biochemical and biomechanical signals [43]. Additionally, ECM conveys specific signals to cells resulting in the modulation of basic functions that are important for the early steps of inflammation, particularly the migration of immune cells during tissue inflammation and immune cell differentiation [44]. These functions of ECM are believed to be mediated primarily by integrins under the family of cell surface receptors [45]. In support, Kroll et al. [46] and Englund et al. [47] reported that platelet membranes, such as GpIb and GpIX, when bound to the von Willebrand factor (vWF), would help transmit signals to the platelet that leads to platelet activation and adhesion. Whereas, the platelet glycoprotein (GP) Ib-IX-V complex is responsible for platelet rolling and adhesion to the site of injury [48]. As such, the literature suggests that the upregulation of integrin subunit beta 4 (ITGB4), GpV, GpIba, and GpIX in this study might be involved in the immune-related functions of ECM.

Fascinatingly, enrichment of ECM receptor pathways supported the succeeding enriched pathways, such as the hematopoietic cell lineage and platelet activation pathways. In a study by Klein [49], it was reported that the ECM matrix molecules (collagen, proteoglycans, and glycoproteins) are part of the bone marrow microenvironment that plays a very significant role in promoting hematopoietic cell proliferation and differentiation. Thus, the upregulation of all DEGs under the hematopoietic cell lineage pathway (GP5, GP1BA, CD24, GP9) supports the observed upregulation of some DEGs under the ECM pathway.

On the other hand, the platelet activation pathway, which are believed to be related to ECM glycoprotein, was reported to be triggered during viral antigen-antibody complexes, from which virus-induced platelet activation can modulate platelet count that help shape immune response through their released products that suppressed infection [50]. Under this pathway, there were four identified upregulated DEGs, namely, GP5, P2RX1, GP1BA, and GP9, with only MAPK12 as down-regulated.

Another important enriched pathway is the ATP-binding cassette (ABC) transporter, which purportedly plays a crucial role in adaptive immunity by its ability to shuttle degrade proteasomal products into the endoplasmic reticulum (ER), which then loaded to MHC class I before antigen presentation on the cell surface [51,52]. In the study of Hinz and Tampé [53], it was also reported that transporters associated with antigen processing (TAP) could be challenged with a number of viral factors, which prevent antigen translocation and loading MHC class I in virally infected cells. Thus, this literature suggests that the upregulation of ABCB11 (ABC transporter pathways) previously observed in this study was associated with the development of adaptive immunity against BVDV Type I. 

Another interesting pathway that tended to be significantly enriched was the type 1 diabetes mellitus pathway. This information catches the attention of researchers due to a previous report that cattle infected with the BVD-mucosal disease virus can induce insulin-dependent diabetes mellitus [54]. 

## 5. Conclusions

The results of this study showed significantly identified DEGs under different immune-related gene ontologies and signaling pathways in response to BVDV type 1 antigen. These observed findings will surely provide assistance by enlightening end-users and other researchers on the changes happening in the animal immune system brought by vaccination. In addition, the potential inclusion of DEGs to animal improvement programs, such as breeding, selection, and genetic manipulation techniques will surely help improve the efficacy of the vaccine. Furthermore, the DEGs, annotation, and pathways identified in this study can be utilized for future studies concerning the immune response of cattle to vaccines.

## Figures and Tables

**Table 1:** Summary of the mapping information for each sample.

Sample ID	Total Raw Reads	Total Clean Reads	GC (%)	Q30 (%)	No. of Processed Reads	No. of Mapped Reads	No. of Unmapped Reads
13064	1.13E + 10	1.12E + 08	45.52	95.28	1.11E + 08	1.08E + 08	2.32E + 06
13083	9.59E + 09	9.50E + 07	45.73	95.27	9.40E + 07	9.12E + 07	2.74E + 06
13090	9.77E + 09	9.67E + 07	45.22	94.65	9.55E + 07	9.38E + 07	1.70E + 06
14010	8.52E + 09	8.44E + 07	45.00	95.31	8.36E + 07	8.17E + 07	1.82E + 06
14017	9.38E + 09	9.29E + 07	45.99	95.30	9.19E + 07	8.93E + 07	2.55E + 06
14107 *	9.67E + 09	9.58E + 07	45.03	95.22	9.47E + 07	9.27E + 07	2.02E + 06
15060 *	8.31E + 09	8.22E + 07	45.54	95.08	8.13E + 07	7.96E + 07	1.62E + 06
15071 *	9.01E + 09	8.92E + 07	45.87	95.23	8.83E + 07	8.18E + 07	6.37E + 06
15083 *	8.37E + 09	8.29E + 07	44.48	95.19	8.20E + 07	8.02E + 07	1.77E + 06
15094 *	7.42E + 09	7.35E + 07	44.86	94.98	7.26E + 07	7.07E + 07	1.92E + 06

* Animals belongs to high immune responses and BVDV type I antibody group.

**Table 2:** List of significantly enriched Kyoto Encyclopedia of Genes and Genomes (KEGG) pathways associated with immune response.

	Pathways	ID	DEGs No.	*p*-Value	Up-Regulated Genes	Down-Regulated Genes
1	Platelet activation	bta04611	5	1.34E-02	GP5, P2RX1, GP1BA, GP9	MAPK12
2	Cytokine-cytokine receptor interaction	bta04060	6	2.07E-02	IL18, IL1RAP	CCR8, CCL3, IL20RA,TGFB2
3	ECM-receptor interaction	bta04512	4	2.52E-02	GP5, GP1BA, GP9	ITGB4
4	Hematopoietic cell lineage	bta04640	4	2.91E-02	GP5, GP1BA, CD24, GP9	-
5	ABC transporters	bta02010	3	3.73E-02	ABCB11	LOC100296627, CFTR
6	Type I diabetes mellitus	bta04940	2	5.48E-02	BOLA, PTPRN2	-

ECM—Extra Cellular Matrix, ABC—ATP Binding Cassette
